# Recent Progress in Antimicrobial Nanomaterials

**DOI:** 10.3390/nano10112315

**Published:** 2020-11-23

**Authors:** Ana Maria Díez-Pascual

**Affiliations:** Department of Analytical Chemistry, Physical Chemistry and Chemical Engineering, Faculty of Sciences, Institute of Chemistry Research “Andrés M. del Río” (IQAR), University of Alcalá, Ctra. Madrid-Barcelona, Km. 33.6, 28871 Alcalá de Henares, Madrid, Spain; am.diez@uah.es; Tel.: +34-918-856-430

Bacterial infections are a well-known and serious problem in numerous areas of everyday life, causing death, pain, and huge added costs to healthcare worldwide. They also cause major issues in many other industries, such as textiles, water treatment, marine transport, medicine, and food packaging. Despite strong efforts by academic researchers and industries, a universal solution for controlling bacterial adhesion and proliferation has not yet been found. Over the last years, many novel antibacterial nanomaterials have been developed, and some of them are already applied in hospitals and public buildings. This Special Issue, with a collection of nine original contributions and two reviews, provides selected examples of the latest advances in the field of antibacterial nanomaterials and their applications in various fields. 

Recent advances in nanoscience and nanotechnology have led to the development of advanced functional nanomaterials with unique chemical and physical properties. The large surface-area-to-volume ratio of nanoparticles (NPs) opens many possibilities for developing bactericidal agents to treat deadly microbial infections. In particular, metal and metal-oxide NPs have attracted great attention as promising candidates for antibacterial agents [1,2]. The key mechanisms for the antibacterial activities of these NPs include: (a) oxidative stress due to reactive oxygen species (ROS) generation [3], in which the oxidation in bacteria cells induces peroxidation of the lipid membrane, thus destructing proteins and DNA.;(b) the release of metal ions release from metal or metal-oxide NPs penetrating over bacteria cell walls that directly interact with amino and carboxylic acid groups of proteins and nucleic acids, resulting in cell death [4]; (c) membrane disruption due to accumulation of the NPs at the bacterial membrane followed by NP internalization. 

Silver nanoparticles (AgNPs) have been applied as antibacterial agents for textile fabrics, healthcare products, cosmetics, coatings, and wound dressings, owing to their effective bactericidal action. Furthermore, they are employed in clinical practice for an extensive range of treatments, such as burns, chronic ulcers, and diabetic wounds that have developed antibiotic resistance. In addition to anti-inflammatory effects, AgNPs-treated wounds have revealed abundant collagen deposition able to accelerate wound healing [5]. However, AgNPs are toxic for several human cell lines and induce dose-, size-, and time-dependent cytotoxicity, especially those with sizes of ≤10 nm [6]. To overcome these disadvantages, their immobilization onto various supporting materials, such as metal oxides, activated carbon, graphene oxide, polymers, etc., have been investigated [7]. The modification of AgNPs with titanate nanotubes (TNT) leads to changes in their physicochemical characteristics, such as size, shape, stability, and oxidation state, leading to improved antibacterial, photocatalytic, and catalytic activities. Immobilization onto polysaccharides such as carboxymethyl-cellulose (CMC) with different degrees of substitution and molecular weight has also been reported [8], and it was found that the particle size distribution and morphology of AgNPs are conditioned by the number of functional groups available for their immobilization. Accordingly, smaller particle sizes were obtained for CMC with a higher degree of substitution, resulting in increased antibacterial activity and cytotoxicity of the samples.

Compared to other types of nanoparticles, titanium dioxide (TiO_2_) is particularly attractive for photocatalytic bactericidal activity, owing to its somewhat low cost, natural abundance, and improved chemical stability [9]. It is an n-type semiconductor due to the presence of oxygen vacancies that favor the formation of Ti^3+^ centers, acting as electron donors [10]. Furthermore, these vacancies can influence charge transport and electron–hole recombination processes by trapping charge carriers in the defect sites. To achieve antibacterial inactivation under visible light, TiO_2_ NPs can be doped with metal and nonmetal elements, modified with carbonaceous nanomaterials, coupled with other metal-oxide semiconductors, or deposited onto fibrous materials [11,12]. The modification of TiO_2_ NPs with carbon-based nanomaterials, such as nanotubes or graphene, also results in efficient ROS formation under visible-light irradiation [13]. By incorporating TiO_2_ NPs into polymers such as chitosan or epoxidized vegetable oils [14], the resulting polymer nanocomposites exhibit excellent antimicrobial properties that can have applications in fruit/food wrapping films, self-cleaning fabrics, medical scaffolds, antimicrobial coatings, and wound dressings.

Copper-containing compounds, such as CuSO_4_ and Cu(OH)_2_, are used as conventional antibacterial agents. In addition, aqueous copper solutions, complex copper species, or copper-containing polymers are used as antifungal compounds. On the other hand, liposomes are nanovesicles made with phospholipids traditionally used as delivery vehicles because phospholipids facilitate cellular uptake. Their carrier capacity and hydrophilic/hydrophobic balance are beneficial for developing hybrid nanostructures based on metallic NPs. Thus, with the aim to improve the effectiveness of traditional bactericide agents, nanovesicular systems have been loaded with Cu NPs electrosynthesized in organic media [15]. The nanovesicles have been synthesized by the thin-film hydration technique in aqueous media, using phosphatidylcholine and cholesterol as membrane stabilizers. Several quaternary ammonium salts were tested as stabilizing surfactants for the synthesis and insertion of CuNPs. These are attached mainly to the membrane, probably due to the attraction of their hydrophobic shell to the phospholipid bilayers. It was found that the stability of the liposomes increased upon increasing NP loading, signifying a charge-stabilization effect in a novel material that can fight against antibiotic-resistant biofilms. The use of nanovesicles is of great interest since their size is in the order of 100 to 1000 nm, whereas safety regulations apply for ultrafine NPs [16].

Zinc oxide (ZnO) nanostructures are widely used materials capable of antimicrobial action [17]. With a wide bandgap of 3.4 eV and large exciton-binding energy of 60 meV at room temperature, they are widely used for optical devices [18]. These environmentally friendly materials possess a large volume-to-area ratio, crystalline structure, radiation hardness, good mechanical properties, and high thermal conductivity and are highly suitable as catalysts, gas sensors, or reinforcing fillers in polymers [19]. They can be obtained by several methods, including physical and chemical approaches. Bearing in mind the recent growth in environmentally friendly and low-cost synthetic routes for nanomaterial synthesis, electrochemical techniques represent a valid alternative to biogenic synthesis. In this regard, the aqueous electrosynthesis of ZnO nanomaterials (both rod-like and flower-like structures) with different aspect ratios based on the use of alternative stabilizers such as benzyl-hexadecyl-dimethylammonium chloride (BAC) and poly-diallyl-(dimethylammonium) chloride (PDDA) has been reported [20]. The combination of UV–vis, FTIR, and XPS spectroscopies demonstrated the whole conversion of the raw colloidal materials into stoichiometric ZnO species with moderate morphological modification. Both BAC- and PDDA-modified nanomaterials showed a strong antimicrobial efficacy against *B. subtilis*, as demonstrated by agar diffusion tests. This approach is an efficient alternative to current methodologies to produce elongated ZnO nanomaterials in an aqueous solution with cationic capping agents, leading to higher yields and milder preparation conditions. Application of these ZnO nanostructures in transistor devices (PDDA-capped) and for cultural heritage preservation (BAC-capped) is foreseen.

Layer-by-layer (LbL) assemblies, based on the alternated adsorption of oppositely charged compounds, is a versatile approach that allows control at the nanoscale [21]. A wide range of LbL antimicrobial coatings comprising polymers, nanoparticles, enzymes, peptides, biological molecules, and antibiotics as building units have been reported [22]. Their antimicrobial action is based on bioadhesion resistance, contact-killing, release-killing, or a combination of such mechanisms [23].

LbL assemblies comprising poly(allylamine hydrochloride) and poly(sodium 4-styrene sulfonate) showed significant antimicrobial activity via contact killing, and poly (L-lysine)/poly (L-glutamic acid) multilayers with the top bilayers bearing the pegylated polyanion drastically suppressed the adsorption of *E. coli* [24]. LbL assemblies comprising two naturally occurring antimicrobials, lysozyme, and chitosan, together with Nafion, a synthetic ionomer-bearing hydrophilic sulfonic acid group, has recently been reported [25]. Owing to its chemical composition, Nafion forms proton-exchange membranes with utmost structural and chemical stability highly suitable for fuel cell applications. Although the surface charges of Nafion were neutralized and even overcompensated by the adsorption of positively charged molecules, the coatings displayed noticeable antimicrobial activity against *E. coli* and *S. aureus*. It is envisaged that the synergistic effect of Nafion and conventional antimicrobial agents can generate highly effective platform coatings with enhanced bactericidal action. 

Polyhydroxyalkanoates (PHAs) are a class of biocompatible and biodegradable polymers belonging to the family of natural polyesters synthesized by bacterial fermentation from renewable resources such as cane sugar [26], widely used in a variety of applications ranging from nanotechnology, medical, tissue engineering, and packing industries [27]. However, PHAs possess limited applications in the biomedical field due to their brittleness and poor mechanical properties. To improve the mechanical and thermal properties of PHAs, they can be copolymerized with different monomers such as 3-hydroxyhexanoate (HHx), providing better flexibility and biodegradability compared to raw PHAs [28]. Furthermore, blending with other biopolymers such as chitosan (Ch) can lead to improved physicochemical properties [29].

Other natural compounds that have a low environmental impact are also receiving widespread attention. In particular, plant-derived bioactive substances have been applied as natural preservatives (e.g., essential oils) in the food industry due to their antifungal properties [14]. Eugenol (1,2-methoxy-4-(2-propenyl)-phenol), a main component of the herbal oil from basil, has been categorized as GRAS (generally recognized as safe) food additive since it is beneficial for the food field due to its antimicrobial properties against a comprehensive range of microorganisms [30]. However, eugenol is highly volatile and has poor water solubility, which limits its applications. Caseins, the main component of milk proteins in bovine milk, is a cheap and commercially available food-grade additive. The amphiphilic nature of caseins makes them suitable for encapsulating compounds of poor water solubility. In this regard, eugenol-entrapped casein nanoparticles have been prepared via a low-energy and simple self-emulsifying technique [31]. A mass ratio of 5:1 of caseins/eugenol yielded the best encapsulation efficiency and stability. This encapsulation with casein noticeably enhances the antifungal efficacy against anthracnose. These results indicate that EC-NPs nanoparticles could be used as an economical and simple-manufactured preservative for postharvest fruits against microbial spoilage.

On the other hand, hexagonal boron nitride (hBN) nanostructures exhibit comparable or even better properties than their carbon counterparts and are stable under oxidative conditions up to 1000 °C. They display a very high Young’s modulus (up to 1.3 TPa), piezoelectricity, hydrogen storage capacity, superhydrophobicity, lubricant behavior, and good biocompatibility, which makes them suitable for biomedical applications including drug delivery, biosensors, biomaterials, and neutron capture therapy [32]. The potential antibacterial effect and cell viability efficacy of PHA/Ch-hBN nanocomposites loaded with three different concentrations of hBN nanoparticles have been recently investigated [33]. Nanocomposites were prepared through a simple solvent casting technique. The fabricated PHA/Ch-hBN nanocomposites demonstrated effective antimicrobial against *S. Aureus* and *E. Coli*, and good biocompatibility properties that would be suitable for biomedical applications. 

Synthetic bactericidal patterned surfaces that are capable of killing the bacteria via mechanical mechanisms have recently been developed via nano-/microfabrication techniques [34]. Different design parameters are known to affect the bactericidal activity of nanopatterns. Evaluating the effects of each parameter, isolated from the others, requires systematic studies. A recent article has evaluated the influence of the interspacing and disordered arrangement of nanopillars on the bactericide properties of nanopatterned surfaces [35]. Electron beam induced deposition (EBID) was used to manufacture the nanopatterns with accurately controlled dimensions as well as disordered versions of them. The killing efficiency of the nanopatterns against *S. Aureus* increased by decreasing the interspace, achieving the highest efficiency on the nanopatterns with 100 nm interspacing. In contrast, the disordered nanopatterns did not influence the killing efficiency significantly, as compared to their ordered correspondents. Thus, optimizing the design of nanopatterns should focus on the interspacing as an important parameter affecting the bactericidal properties. 
